# Supplementary material for: Characterization of a Novel Mitovirus of the Sand Fly Lutzomyia longipalpis Using Genomic and Virus–Host Interaction Signatures
Source: Viruses. 2020 Dec 23;13(1):9. doi: 10.3390/v13010009 (PMC7822452; doi:10.3390/v13010009)
Supplement: Supplementary file 1 [file viruses-13-00009-s001.zip › Fonseca et al - Supplementary Material.docx]

**SUPPLEMENTARY MATERIAL**

Characterization of a novel mitovirus of the sand fly *Lutzomyia longipalpis* using genomic and virus-host interaction signatures

**Paula Fonseca^1^, Flavia Ferreira^2^, Felipe da Silva^3^, Liliane Santana Oliveira^4,7^, João Trindade Marques^2,3,5^, Aristóteles Goes-Neto^1,3^, Eric Aguiar^3,6,*,†^ and Arthur Gruber^4,7,8*,†^**

^1^ Department of Microbiology, Instituto de Ciências Biológicas, Universidade Federal de Minas Gerais, Belo Horizonte MG, 30270-901, Brazil; [camargos.paulaluize@gmail.com](mailto:camargos.paulaluize@gmail.com)

^2^ Department of Biochemistry and Immunology, Instituto de Ciências Biológicas, Universidade Federal de Minas Gerais, Belo Horizonte, MG, 30270-901, Brazil; [fvianaferreira@gmail.com](mailto:fvianaferreira@gmail.com)

^3^ Bioinformatics Postgraduate Program, Instituto de Ciências Biológicas, Universidade Federal de Minas Gerais, Belo Horizonte, MG, 30270-901, Brazil; [felselva@gmail.com](mailto:felselva@gmail.com), arigoesneto@gmail.com

^4^ Bioinformatics Postgraduate Program, Universidade de São Paulo, São Paulo, SP, 05508-000, Brazil; [liliane.sntn@gmail.com](mailto:liliane.sntn@gmail.com)

^5^ Université de Strasbourg, CNRS UPR9022, Inserm U1257, 67084 Strasbourg, France; [jtmarques2009@gmail.com](mailto:jtmarques2009@gmail.com)

^6^ Department of Biological Science (DCB), Center of Biotechnology and Genetics (CBG), State University of Santa Cruz (UESC), Rodovia Ilhéus-Itabuna km 16, Ilhéus, BA, 45652-900, Brazil; [ericgdp@gmail.com](mailto:ericgdp@gmail.com)

^7^ Department of Parasitology, Instituto de Ciências Biomédicas, Universidade de São Paulo, São Paulo, SP, 05508-000, Brazil; [argruber@usp.br](mailto:argruber@usp.br)

^8^European Virus Bioinformatics Center, Leutragraben 1, Jena, 07743, Germany

* Correspondence: [ericgdp@gmail.com](mailto:ericgdp@gmail.com) (EA) and [argruber@usp.br](mailto:argruber@usp.br) (AG).

† Both corresponding authors contributed equally to this work.

**Figure S1.** Percentage of vFam domains found in the long RNA library of *Lutzomyia longipalpis*. Functional annotation of the contigs obtained by progressive assembly using vFam models as seeds is listed in the Supplementary Table S5.

**Figure S2. Comparative analysis of dinucleotide frequencies of the novel virus and putative host genomes.** Correlation plot calculated with corrplot program and based on dinucleotide odds ratio of the *Lutzomyia longipalpis mitovirus 1* (Lul-MV-1), other mitoviruses and mitochondrial genomes (MT) of some fungi and insects. Clustering was performed using K-means method.


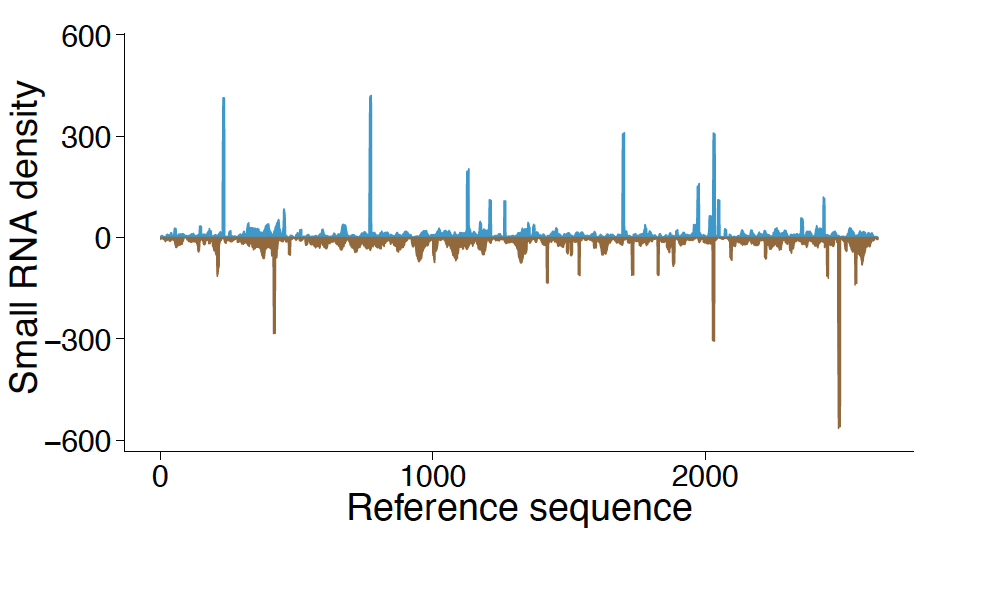


**Figure S3. Distribution of virus-derived small RNAs mapping on the *Lutzomyia longipalpis mitovirus 1* (Lul-MV-1) genome.** Blue and brown colors indicate density of small RNA reads mapped to the positive and negative strands of the viral genome, respectively.

**Figure S4. Sequence alignment of a fragment of the RdRp gene.** Primers based on the assembled sequence of Lul-MV-1 were designed and used to amplify a 509-bp target of the RdRp gene using total RNA from pools of *Lu. longipalpis* individuals maintained in a laboratory colony. PCR fragments were submitted to Sanger sequencing and aligned to the Lul-MV-1 sequence. The query corresponds to the nucleotide sequence obtained from one of the pools and the subject to the Lul-MV-1 sequence.

**Table S1.** Public RNA libraries of *Lu. longipalpis* analyzed in this study.

| **SRA ID** | **Strategy** | **Number of reads** |
| --- | --- | --- |
| SRR535766 | long RNAs | 24,496,036 |
| SRR535767 | long RNAs | 18,577,781 |
| SRR1803384 | small RNAs | 10,679,412 |
| SRR1803385 | small RNAs | 9,463,241 |
| SRR1803386 | small RNAs | 8,109,613 |
| SRR5224248 | small RNAs | 8,587,131 |
| SRR5224249 | small RNAs | 8,500,448 |
| SRR6429752 | small RNAs | 8,766,757 |

**Table S2.** Public protein sequences used in this work.

| **Organism** | **Accession number** |
| --- | --- |
| *Aedes japonicus narnavirus 1* | QIP67846 |
| *Alternaria arborescens mitovirus 1* | YP_009270635 |
| *Aspergillus fumigatus mitovirus 1* | AXE72932 |
| *Barns Ness breadcrumb sponge narna-like virus 4* | ASM94069 |
| *Beihai narna-like virus 9* | YP_009333316 |
| *Beihai narna-like virus 18* | YP_009333315 |
| *Binucleate Rhizoctonia mitovirus K1* | YP_009165597 |
| *Buergenerula spartinae mitovirus 1* | AHY03257 |
| *Cassava virus C* | YP_003104770 |
| *Coquillettidia venezuelensis narnavirus 2* | QBA55487 |
| *Cryphonectria parasitica mitovirus 1* | NP_660174 |
| *Culex narnavirus 1* | QBR53296 |
| *Enterobacteria phage MS2* | YP_009640127 |
| *Epirus cherry virus* | YP_002019754 |
| *Escherichia virus BZ13* | NP_040755 |
| *Fusarium poae mitovirus 4* | YP_009272901 |
| *Grapevine-associated narnavirus 1* | CEZ26304 |
| *Haverford narna-like virus* | QIJ70061 |
| *Hubei narna-like virus 3* | YP_009337787 |
| *Hubei narna-like virus 13* | YP_009337805 |
| *Lutzomyia longipalpis mitovirus 1* (Lul-MV-1) | BK013136 |
| *Mitovirus AEF-2013* | AGW51760 |
| *Mitovirus JS3* | QHA33929.1 |
| *Narnaviridae* environmental sample | AJT39596 |
| *Ochlerotatus-associated narna-like virus 1* | AGW51766 |
| *Ochlerotatus-associated narna-like virus 2* | AGW51768 |
| *Ophiostoma mitovirus 4* | NP_660179 |
| *Ourmia melon virus* | YP_002019757 |
| *Plasmopara viticola lesion-associated mitovirus 56* | QIR30279 |
| *Plasmopara viticola lesion-associated narnavirus 17* | QIR30296 |
| *Plasmopara viticola lesion-associated mitovirus 23* | QIR30246 |
| *Plasmopara viticola lesion-associated mitovirus 39* | QIR30262 |
| *Psorophora varipes narnavirus* | QBA55486 |
| *Rhizoctonia solani mitovirus 28* | QDW65418 |
| *Rhizoctonia solani mitovirus 30* | QDW65420 |
| *Rhizopus microsporus 20S narnavirus* | QBC65280 |
| *Rhizopus microsporus 23S narnavirus* | QBC65281 |
| *Saccharomyces 20S RNA narnavirus* | NP_660178 |
| *Saccharomyces 23S RNA narnavirus* | AAC98708 |
| *Sclerotinia sclerotiorum mitovirus 1 HC025* | YP_009121785 |
| *Sclerotinia sclerotiorum mitovirus 1-A2* | AWY10962 |
| *Setosphaeria turcica mitovirus 1* | AZT88625 |
| *Shahe narna-like virus 6* | APG77166 |
| *Soybean leaf-associated mitovirus 2* | ALM62242 |
| *Tuber excavatum mitovirus* | AEP83726 |
| *Tynnyfer narna-like virus* | QIJ70064 |
| *Wenling narna-like virus 9* | YP_009337200 |
| *Wuhan insect virus 18* | YP_009342440 |
| *Zhejiang mosquito virus 3* | YP_009333331.1 |

**Table S3.** Public genome sequences used in this work.

| **Sample source/organism** | **Accession number** |
| --- | --- |
| **Mitochondrial genome** |  |
| *Aedes aegypti* | NC_035159 |
| *Anopheles atroparvus* | NC_028213 |
| *Aspergillus fumigatus* | NC_017016 |
| *Beauveria bassiana* | NC_010652 |
| *Cordyceps militaris* | KP719097 |
| *Culex quinquefasciatus* | NC_014574 |
| *Cyberlindnera jadinii* | NC_022163 |
| *Lutzomyia longipalpis* | BK014371 |
| *Metarhizium anisopliae* | NC_008068 |
| [*Nyssomyia umbratilis*](https://www.ncbi.nlm.nih.gov/nuccore/KP702938.1/) | KP702938 |
| *Plasmopara viticola* | NC_045922 |
| *Saccharomyces cerevisiae* | NC_027264 |
| *Sclerotinia sclerotiorum* | NC_035155 |
| **Viral genome** |  |
| *Aedes japonicus narnavirus 1* | MK984721 |
| *Alternaria arborescens mitovirus 1* | NC_030747 |
| *Barns Ness breadcrumb sponge narna-like virus 4* | MF190030 |
| *Binucleate Rhizoctonia mitovirus K1* | NC_027921 |
| *Buergenerula spartinae mitovirus 1* | KJ485703 |
| *Colletotrichum falcatum mitovirus 1* | MK279482 |
| *Coquillettidia venezuelensis narnavirus 2* | MK285334 |
| *Cryphonectria parasitica mitovirus 1* | NC_004046 |
| *Culex narnavirus 1* | MK628543 |
| *Entomophthora muscae mitovirus 6* | BK010734 |
| *Entomophtohora muscae mitovirus 7* | MK682519 |
| *Erysiphe necator mitovirus 1* | NC_037054 |
| *Erysiphe necator mitovirus 3* | NC_037056 |
| *Fusarium poae mitovirus 4* | NC_030864 |
| *Gigaspora margarita mitovirus 1* | NC_040702 |
| *Grapevine associated narnavirus 1* | LN827948 |
| *Haverford narna-like virus* | MT129703 |
| *Leptosphaeria biglobosa mitovirus 1* | NC_040819 |
| *Lutzomyia longipalpis mitovirus 1* (Lul-MV-1) | BK013136 |
| *Macrophomina phaseolina mitovirus 3* | KT823703 |
| *Narnaviridae* environmental sample clone | KP642119 |
| *Ochlerotatus-associated narna-like virus 2* | KF298276 |
| *Plasmopara viticola lesion-associated mitovirus56* | MN539817 |
| *Psorophora varipes narnavirus* | MK285336 |
| *Rhizopus microsporus 20S narnavirus* | MK204624 |
| *Sclerotinia sclerotiorum mitovirus 1* | NC_026510 |
| *Sclerotinia sclerotiorum mitovirus 1-A2* | MF444234 |
| *Setosphaeria turcica mitovirus 1* | MK279486 |
| *Shahe narna-like virus 6* | KX883554 |
| *Soybean leaf-associated mitovirus 2* | KT598239 |
| *Tuber aestivum mitovirus* | NC_015629 |
| *Tuber excavatum mitovirus* | JN222389 |
| *Tynnyfer narna-like virus* | MT129705 |
| *Wenling narna-like virus 9* | NC_033064 |
| *Zhejiang mosquito virus 3* | MF176365 |

**Table S4.** Oligonucleotides designed and used in this study.

| **Forward primer (5`-3`)** | **Reverse primer (5`-3`)** | **Amplicon size (bp)** |
| --- | --- | --- |
| GGAAGATCCATTAGGTCCAG | CAGGTACACGAAGAGTCCTA | 509 |
|  |  |  |
|  |  |  |

**Table S5.** Functional annotation of the sequences used to build the original vFam* models utilized as seeds for progressive assembly with GenSeed-HMM program.

| **Profile HMM** | **Functional annotation** |
| --- | --- |
| vFam_2927 | DNA-directed RNA polymerase II subunit 1 |
| vFam_2543 | DNA-directed RNA polymerase II subunit RPB5 |
| vFam_5985 | RNA-dependent DNA polymerase |
| vFam_69 | DNA polymerase |
| vFam_37 | Reverse transcriptase |
| vFam_387 | Capsid protein |
| vFam_3987 | Reverse transcriptase |
| vFam_3011 | Reverse transcriptase |
| vFam_561 | Nucleoprotein N |
| vFam_1529 | Nucleoprotein N |
| vFam_5143 | DNA-directed RNA polymerase subunit alpha |
| vFam_3906 | DNA-directed RNA polymerase subunit alpha |
| vFam_571 | RNA-dependent RNA polymerase |
| vFam_1961 | RNA-dependent RNA polymerase |
| vFam_1272 | RNA-dependent RNA polymerase |
| vFam_87 | Capsid protein |
| vFam_1118 | Capsid protein |
| vFam_254 | DNA polymerase |

*vFAM database of profile HMMs (<http://derisilab.ucsf.edu/software/vFam/>).

**Table S6.** Functional annotation of the contigs obtained by progressive assembly using vFam models as seeds.

| **vFam seed** | **Number of assembled contigs** | **Function annotation of the coded protein** |
| --- | --- | --- |
| vFam_2927 | 4 | Contig 1 – RNA polymerase 2 |
| vFam_2543 | 1 | Contig 1 – DNA-dependent RNA polymerase |
| vFam_5985 | 71 | Contig 1 – Reverse transcriptase  Contig 3 – RNA-dependent DNA polymerase |
| vFam_69 | 8 | Contig 1 – DNA polymerase subunit epsilon  Contig 2 – DNA polymerase delta |
| vFam_37 | 66 | Contig 1 – Reverse ribonuclease integrase |
| vFam_387 | 18 | Contig 1 – Reverse transcriptase  Contig 2 – DNA helicase |
| vFam_3987 | 34 | Contig 1 – Reverse ribonuclease integrase |
| vFam_3011 | 40 | Contig 1 – Reverse ribonuclease integrase |
| vFam_5143 | 7 | Contig 1 – DNA-dependent RNA polymerase |
| vFam_3906 | 5 | Contig 1 – DNA-dependent RNA polymerase |
| vFam_571 | 1 | Contig 1 – RNA-dependent RNA polymerase |
| vFam_1961 | 3 | Contig 1 – RNA-dependent RNA polymerase |
| vFam_1272 | 3 | Contig 1 – RNA-dependent RNA polymerase |
| vFam_87 | 2 | Contig 1 – Capsid protein |
| vFam_1118 | 2 | Contig 1 – Capsid protein |
| vFam_254 | 5 | Contig 1 – DNA polymerase |
| vFam_561 | 12 | Contig 1 – Nucleoprotein N |
| vFam_1529 | 6 | Contig 1 – Nucleoprotein N |

**Table S7.** Use of UGA and UGG codons in coding sequences of some viruses and their putative hosts.

| **Accession number** | **Organism/source** | **# of**  **UGA / UGG** | **% of**  **UGA / UGG** | **AT content (%)** |
| --- | --- | --- | --- | --- |
|  | ***Leviviridae*** |  |  |  |
| NC_001426 | *Escherichia virus BZ13* | ***1/26 | 3.7/96.3 | 52.14 |
| NC_001417 | *Escherichia virus MS2* | 0/23 | 0.8/100 | 47.88 |
| NC_000913 | *Escherichia coli* str. K-12 substr. MG1655 | ***167/20886 | 0/100 | 50.80 |
|  | ***Botourmiaviridae/*Ourmia-like viruses** |  |  |  |
| MH192988 | *Aspergillus fumigatus mitovirus 1* | ***1/19 | 5.0/95.0 | 45.02 |
| NC_017016 | *Aspergillus fumigatus* mitochondrion | 68/5 | 93.2/6.8 | 74.52 |
| NC_013111 | *Cassava virus C* | 0/17 | 0/100 | 50.07 |
| NC_011065 | *Epirus cherry virus* | 0/14 | 0/100 | 46.42 |
| NC_011068 | *Ourmia melon virus* | ***1/14 | 6.7/93.3 | 48.47 |
|  | ***Narnaviridae*** |  |  |  |
| MK984721 | *Aedes japonicus narnavirus 1* | 0/11 (RdRp) 0/9 (HR) | 0/100 (RdRp) 0/100 (HR) | 40.83 |
| MF194022 | *Aedes aegypti* mitochondrion | 99/3 | 97.1/2.9 | 78.96 |
| MK628543 | *Culex narnavirus 1* | 0/18 (RdRp) 0/7 (HR) | 0/100 (RdRp) 0/100 (HR) | 38.54 |
| GU188856 | *Culex quinquefasciatus* mitochondrion | 94/5 | 94.9/5.1 | 78.14 |
| KF298276 | *Ochlerotatus-associated narna-like virus 2* | 0/15 (RdRp) 0/13 (HR) | 0/100 (RdRp) 0/100 (HR) | 40.24 |
| MT993477 | *Ochlerotatus caspius* mitochondrion** | 15/0 | 100/0 | 70.66 |
| NC_004051 | *Saccharomyces 20S RNA narnavirus* | ***1/18 | 5.3/94.7 | 41.73 |
| NC_004050 | *Saccharomyces 23S RNA narnavirus* | ***1/20 | 4.8/95.2 | 41.02 |
| MK628543 | *Saccharomyces cerevisiae* mitochondrion | 39/0 | 100/0 | 83.87 |
|  | ***Mitoviridae*** |  |  |  |
| NC_004046 | *Cryphonectria mitovirus 1* | 9/8 | 52.9/47.1 | 63.42 |
| AF456838 | *Cryphonectria parasitica* mitochondrion** | 23/5 | 82.1/17.9 | 67.95 |
| NC_026510 | *Sclerotinia sclerotiorum mitovirus 1* | 10/3 | 76.9/23.1 | 60.24 |
| MF444234 | *Sclerotinia sclerotiorum mitovirus 1-A2* | 8/4 | 66.7/33.3 | 63.37 |
| KT283062 | *Sclerotinia sclerotiorum* mitochondrion | 125/29 | 81.2/18.8 | 69.10 |
| NC_004052 | *Ophiostoma mitovirus 4* | 11/2 | 84.6/15.4 | 73.26 |
| KY084297 | *Ophiostoma novo-ulmi* mitochondrion** | 16/1 | 94.1/5.9 | 76.18 |
| BK013136 | *Lutzomiya longipalpis mitovirus 1* | 11/4 | 73.3/26.7 | 69.74 |
| BK013137 | *Lutzomiya longipalpis* mitochondrion | 99/1 | 99.0/1.0 | 78.07 |
| MN539817 | *Plasmopara viticola associated mitovirus 56* | 11/11 | 50.0/50.0 | 57.53 |
| NC_045922 | *Plasmopara viticola* mitochondrion | ***1/101 | 1.0/99.0 | 76.29 |
| KX883554.1 | *Shahe narna-like virus* | 0/8 | 0/100 | 65.34 |
| NC_033064 | *Wenling narna-like virus 9* | 13/1 | 92.9/7.1 | 63.84 |

*Abbreviations: HR - Hypothetical gene coded in the reverse frame; RdRp – RNA-dependent RNA polymerase. **Incomplete sequence. ***UGA codon used for signaling translation termination.
