# Supplementary material for: Characterization of a Novel Mitovirus of the Sand Fly Lutzomyia longipalpis Using Genomic and Virus–Host Interaction Signatures
Source: Viruses. 2020 Dec 23;13(1):9. doi: 10.3390/v13010009 (PMC7822452; doi:10.3390/v13010009)
Supplement: Supplementary file 1 [file viruses-13-00009-s001.zip › Fonseca et al - Supplementary Material.pdf]

SUPPLEMENTARY MATERIAL

Characterization of a novel mitovirus of the sand fly *Lutzomyia longipalpis* using genomic and virus-host interaction signatures

Paula Fonseca[1], Flavia Ferreira[2], Felipe da Silva[3], Liliane Santana Oliveira[4,7], João Trindade Marques[2,3,5], Aristóteles Goes-Neto[1,3], Eric Aguiar[3,6,*,†] and Arthur Gruber[4,7,8*,†]

[1] Department of Microbiology, Instituto de Ciências Biológicas, Universidade Federal de Minas Gerais, Belo Horizonte MG, 30270-901, Brazil; camargos.paulaluize@gmail.com
[2] Department of Biochemistry and Immunology, Instituto de Ciências Biológicas, Universidade Federal de Minas Gerais, Belo Horizonte, MG, 30270-901, Brazil; fvianaferreira@gmail.com
[3] Bioinformatics Postgraduate Program, Instituto de Ciências Biológicas, Universidade Federal de Minas Gerais, Belo Horizonte, MG, 30270-901, Brazil; felselva@gmail.com, arigoesneto@gmail.com
[4] Bioinformatics Postgraduate Program, Universidade de São Paulo, São Paulo, SP, 05508-000, Brazil; liliane.sntn@gmail.com
[5] Université de Strasbourg, CNRS UPR9022, Inserm U1257, 67084 Strasbourg, France; jtmarques2009@gmail.com
[6] Department of Biological Science (DCB), Center of Biotechnology and Genetics (CBG), State University of Santa Cruz (UESC), Rodovia Ilhéus-Itabuna km 16, Ilhéus, BA, 45652-900, Brazil; ericgdp@gmail.com
[7] Department of Parasitology, Instituto de Ciências Biomédicas, Universidade de São Paulo, São Paulo, SP, 05508-000, Brazil; argruber@usp.br
[8]European Virus Bioinformatics Center, Leutragraben 1, Jena, 07743, Germany
* Correspondence: ericgdp@gmail.com (EA) and argruber@usp.br (AG).
† Both corresponding authors contributed equally to this work.

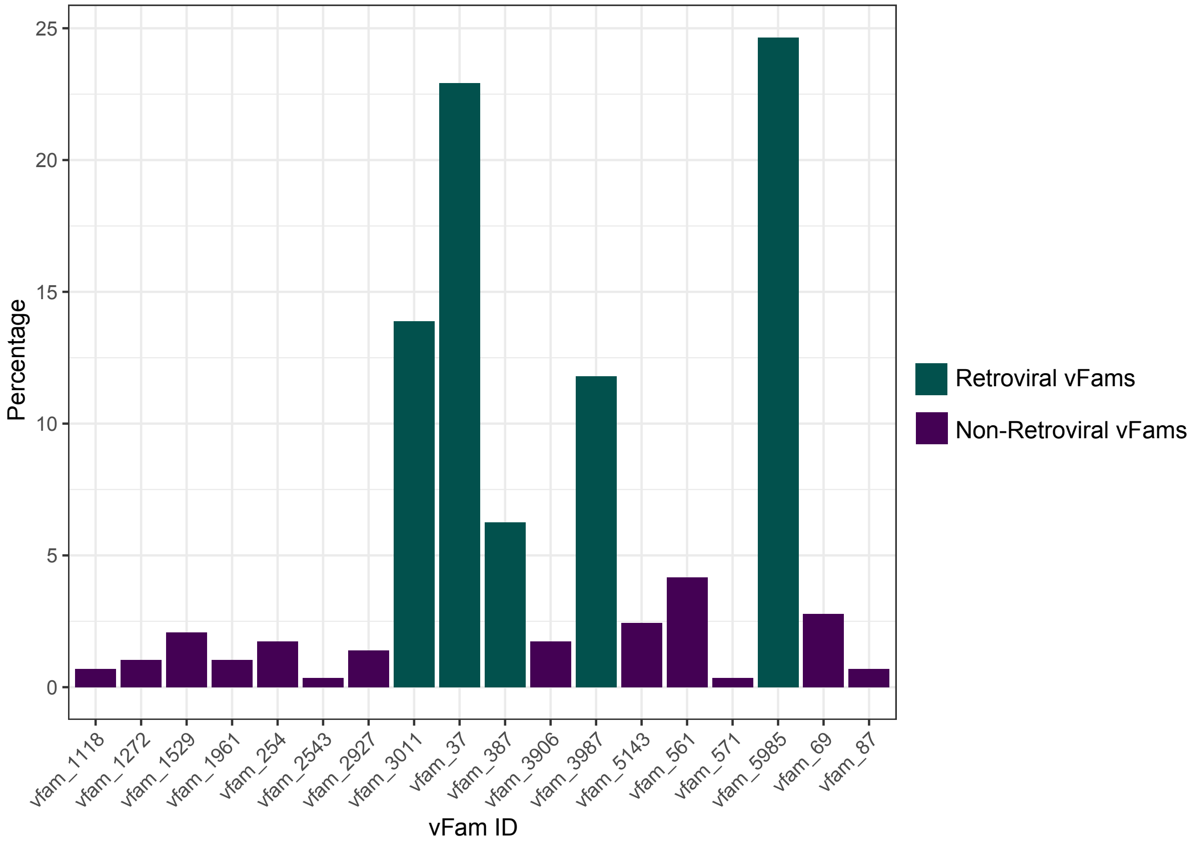

Figure S1. Percentage of vFam domains found in the long RNA library of *Lutzomyia longipalpis*. Functional annotation of the contigs obtained by progressive assembly using `vFam` models as seeds is listed in the Supplementary Table S5.

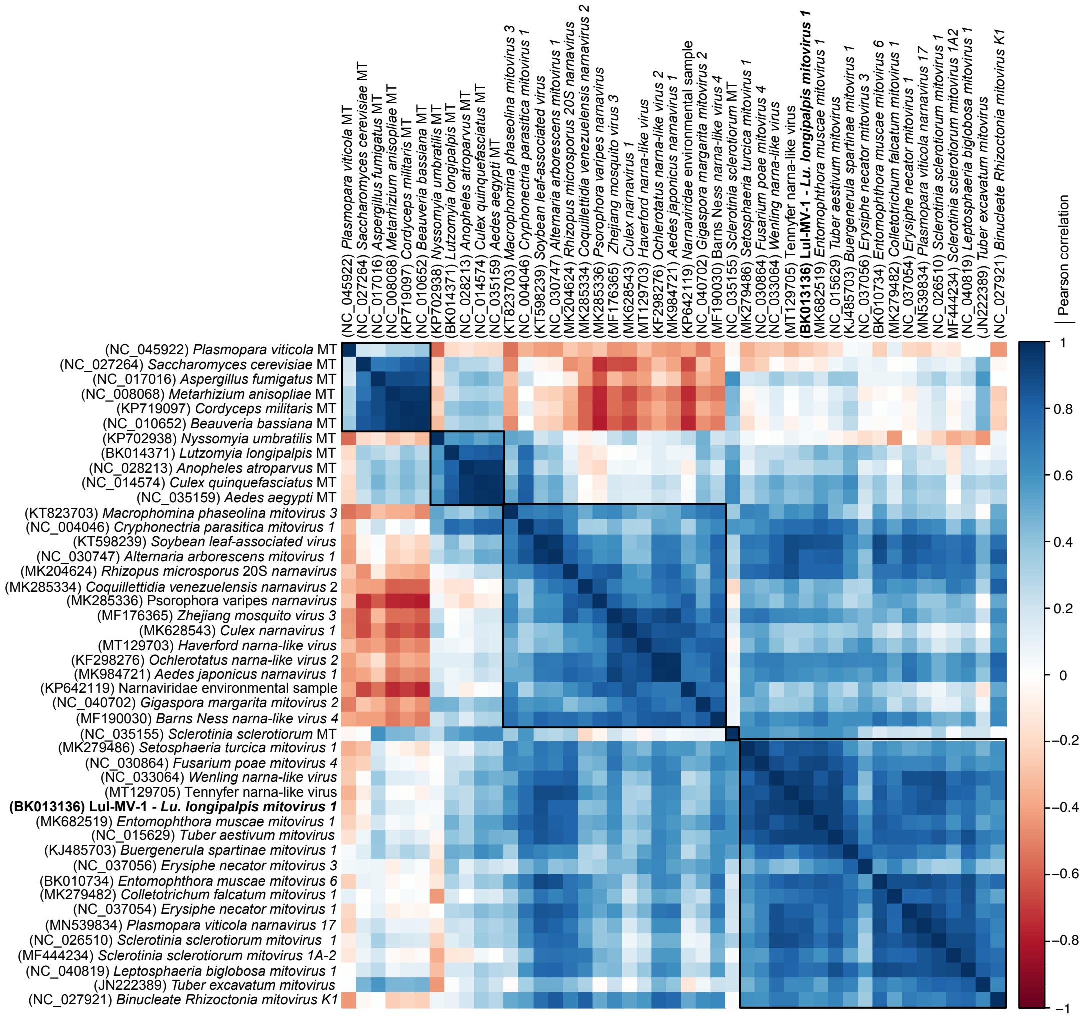

Figure S2. Comparative analysis of dinucleotide frequencies of the novel virus and putative host genomes. Correlation plot calculated with `corrplot` program and based on dinucleotide odds ratio of the *Lutzomyia longipalpis mitovirus 1* (Lul-MV-1), other mitoviruses and mitochondrial genomes (MT) of some fungi and insects. Clustering was performed using K-means method.

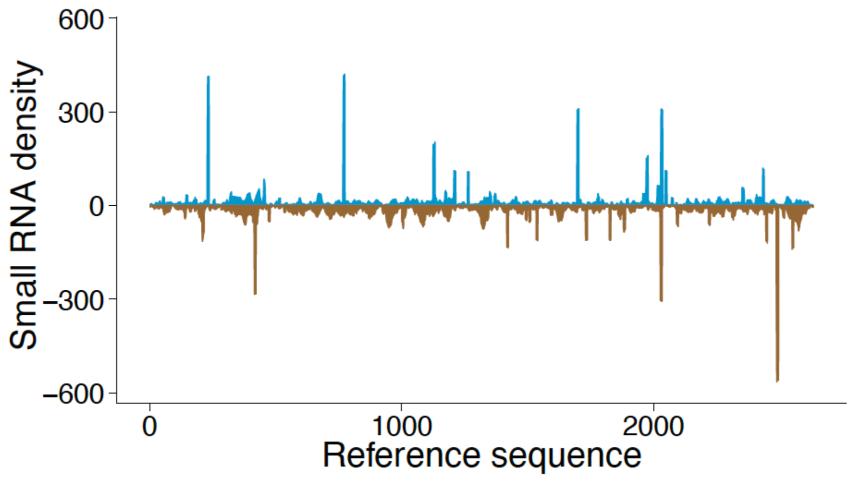

Figure S3. Distribution of virus-derived small RNAs mapping on the *Lutzomyia longipalpis mitovirus 1* (Lul-MV-1) genome. Blue and brown colors indicate density of small RNA reads mapped to the positive and negative strands of the viral genome, respectively.

Sequence ID: Query_137189 Length: 2634 Number of Matches: 1
Range 1: 1926 to 2409

Score	Expect	Identities	Gaps	Strand	Frame
767 bits(415)	0.0()	461/484(95%)	0/484(0%)	Plus/Plus	

Features:

```
Query  1     AGGTCCAGAGGCAGAATATACACTTCCGGTTGGTAAACCAGGAGTATATATTTCACCTAA  60
Sbjct  1926  AGGTCCAGAAGCAGAATATACACTTCCGGTTTGTAAACCAGGAGTATATATTTCGCCTAA  1985

Query  61    GGATTTAATGGAAATTCCTATCTTTCGAAGATCAGCTACTGATTGGACTAACTTCAAGTC  120
Sbjct  1986  AGATTTAATGGAAATTCCTATGTTTTTAAGATCTGCTACTGATTGAACTAACTTCAAGTC  2045

Query  121   ATATATTTTAAGAATAATAATTCCATTCTTAATAATATGGTAAATGTTGGAAAAGGTTAT  180
Sbjct  2046  ATATATTTTAAGAATAATATTTCCATTCTTAATAATATGGTAAATGTTGGTAAAAGATAT  2105

Query  181   TACCTTAACTACAGATACCAAATTAAGAGGAACTGGAGTATTAAACTTTTTATATACTCG  240
Sbjct  2106  AACTTTTACTACAGATACCAAATTAAGAGGAACTGGAGTATTAAACTTTTTATATACTCG  2165

Query  241   AAGTTGGGTTAGGATAATTATCTTATCATAATCTGGAATTTCATACAGTGAAAGAGGATA  300
Sbjct  2166  AAGTTGGGTTAGGATAATCATTTTATCATAATCTGGAATTTCATACAGTGAAAGAGGATA  2225

Query  301   AAGATATGATGGAAAACCATCATTGACCTTTCGTCATCAGACAGATTGGTCAATCTTTAC  360
Sbjct  2226  AAGATATGATGGAAAACCATCATTGACCTTTCGTCATCAGACAGATTGGTCAATCTTTAC  2285

Query  361   CTTTTCACCTGTAAGAAAATACAGTTTGATAAAATTCATATCCTCCTTAAAGCGAGCAAC  420
Sbjct  2286  CTTTTCACCTGTAAGAAAATACAGTTTGATAAAATTTATATCCTCCTTAAATCGAGCAAC  2345

Query  421   AATCATTTCTGGTTGTTGCTTCTTTAAAGATTCCTTCTGAACATGATAGAGTCTATTTAG  480
Sbjct  2346  AATCATTTCTGGTTGTTGTTTCCTTAAGGATTCCTTCTGAACATGATAGAGTCTATTTAG  2405

Query  481   GACT  484
Sbjct  2406  GACT  2409
```

Figure S4. Sequence alignment of a fragment of the RdRp gene. Primers based on the assembled sequence of Lul-MV-1 were designed and used to amplify a 509-bp target of the RdRp gene using total RNA from pools of *Lu. longipalpis* individuals maintained in a laboratory colony. PCR fragments were submitted to Sanger sequencing and aligned to the Lul-MV-1 sequence. The query corresponds to the nucleotide sequence obtained from one of the pools and the subject to the Lul-MV-1 sequence.

Table S1. Public RNA libraries of *Lu. longipalpis* analyzed in this study.

SRA ID	Strategy	Number of reads
SRR535766	long RNAs	24,496,036
SRR535767	long RNAs	18,577,781
SRR1803384	small RNAs	10,679,412
SRR1803385	small RNAs	9,463,241
SRR1803386	small RNAs	8,109,613
SRR5224248	small RNAs	8,587,131
SRR5224249	small RNAs	8,500,448
SRR6429752	small RNAs	8,766,757

Table S2. Public protein sequences used in this work.

Organism	Accession number
Aedes japonicus narnavirus 1	QIP67846
Alternaria arborescens mitovirus 1	YP_009270635
Aspergillus fumigatus mitovirus 1	AXE72932
Barns Ness breadcrumb sponge narna-like virus 4	ASM94069
Beihai narna-like virus 9	YP_009333316
Beihai narna-like virus 18	YP_009333315
Binucleate Rhizoctonia mitovirus K1	YP_009165597
Buergenerula spartinae mitovirus 1	AHY03257
Cassava virus C	YP_003104770
Coquillettidia venezuelensis narnavirus 2	QBA55487
Cryphonectria parasitica mitovirus 1	NP_660174
Culex narnavirus 1	QBR53296
Enterobacteria phage MS2	YP_009640127
Epirus cherry virus	YP_002019754
Escherichia virus BZ13	NP_040755
Fusarium poae mitovirus 4	YP_009272901
Grapevine-associated narnavirus 1	CEZ26304
Haverford narna-like virus	QIJ70061
Hubei narna-like virus 3	YP_009337787
Hubei narna-like virus 13	YP_009337805
Lutzomyia longipalpis mitovirus 1 (Lul-MV-1)	BK013136
Mitovirus AEF-2013	AGW51760
Mitovirus JS3	QHA33929.1
Narnaviridae environmental sample	AJT39596
Ochlerotatus-associated narna-like virus 1	AGW51766
Ochlerotatus-associated narna-like virus 2	AGW51768
Ophiostoma mitovirus 4	NP_660179
Ourmia melon virus	YP_002019757
Plasmopara viticola lesion-associated mitovirus 56	QIR30279
Plasmopara viticola lesion-associated narnavirus 17	QIR30296
Plasmopara viticola lesion-associated mitovirus 23	QIR30246
Plasmopara viticola lesion-associated mitovirus 39	QIR30262
Psorophora varipes narnavirus	QBA55486
Rhizoctonia solani mitovirus 28	QDW65418
Rhizoctonia solani mitovirus 30	QDW65420
Rhizopus microsporus 20S narnavirus	QBC65280
Rhizopus microsporus 23S narnavirus	QBC65281
Saccharomyces 20S RNA narnavirus	NP_660178
Saccharomyces 23S RNA narnavirus	AAC98708
Sclerotinia sclerotiorum mitovirus 1 HC025	YP_009121785
Sclerotinia sclerotiorum mitovirus 1-A2	AWY10962
Setosphaeria turcica mitovirus 1	AZT88625
Shahe narna-like virus 6	APG77166
Soybean leaf-associated mitovirus 2	ALM62242
Tuber excavatum mitovirus	AEP83726
Tynnyfer narna-like virus	QIJ70064
Wenling narna-like virus 9	YP_009337200
Wuhan insect virus 18	YP_009342440
Zhejiang mosquito virus 3	YP_009333331.1

Table S3. Public genome sequences used in this work.

Sample source/organism	Accession number
**Mitochondrial genome**	
Aedes aegypti	NC_035159
Anopheles atroparvus	NC_028213
Aspergillus fumigatus	NC_017016
Beauveria bassiana	NC_010652
Cordyceps militaris	KP719097
Culex quinquefasciatus	NC_014574
Cyberlindnera jadinii	NC_022163
Lutzomyia longipalpis	BK014371
Metarhizium anisopliae	NC_008068
Nyssomyia umbratilis	KP702938
Plasmopara viticola	NC_045922
Saccharomyces cerevisiae	NC_027264
Sclerotinia sclerotiorum	NC_035155
**Viral genome**	
Aedes japonicus narnavirus 1	MK984721
Alternaria arborescens mitovirus 1	NC_030747
Barns Ness breadcrumb sponge narna-like virus 4	MF190030
Binucleate Rhizoctonia mitovirus K1	NC_027921
Buergenerula spartinae mitovirus 1	KJ485703
Colletotrichum falcatum mitovirus 1	MK279482
Coquillettidia venezuelensis narnavirus 2	MK285334
Cryphonectria parasitica mitovirus 1	NC_004046
Culex narnavirus 1	MK628543
Entomophthora muscae mitovirus 6	BK010734
Entomophtohora muscae mitovirus 7	MK682519
Erysiphe necator mitovirus 1	NC_037054
Erysiphe necator mitovirus 3	NC_037056
Fusarium poae mitovirus 4	NC_030864
Gigaspora margarita mitovirus 1	NC_040702
Grapevine associated narnavirus 1	LN827948
Haverford narna-like virus	MT129703
Leptosphaeria biglobosa mitovirus 1	NC_040819
Lutzomyia longipalpis mitovirus 1 (Lul-MV-1)	BK013136
Macrophomina phaseolina mitovirus 3	KT823703
Narnaviridae environmental sample clone	KP642119
Ochlerotatus-associated narna-like virus 2	KF298276
Plasmopara viticola lesion-associated mitovirus56	MN539817
Psorophora varipes narnavirus	MK285336
Rhizopus microsporus 20S narnavirus	MK204624
Sclerotinia sclerotiorum mitovirus 1	NC_026510
Sclerotinia sclerotiorum mitovirus 1-A2	MF444234
Setosphaeria turcica mitovirus 1	MK279486
Shahe narna-like virus 6	KX883554
Soybean leaf-associated mitovirus 2	KT598239
Tuber aestivum mitovirus	NC_015629
Tuber excavatum mitovirus	JN222389
Tynnyfer narna-like virus	MT129705
Wenling narna-like virus 9	NC_033064
Zhejiang mosquito virus 3	MF176365

Table S4. Oligonucleotides designed and used in this study.

**Forward primer (5`-3`)**	**Reverse primer (5`-3`)**	**Amplicon size (bp)**
GGAAGATCCATTAGGTCCAG	CAGGTACACGAAGAGTCCTA	509

Table S5. Functional annotation of the sequences used to build the original `vFam`* models utilized as seeds for progressive assembly with `GenSeed-HMM` program.

Profile HMM	Functional annotation
vFam_2927	DNA-directed RNA polymerase II subunit 1
vFam_2543	DNA-directed RNA polymerase II subunit RPB5
vFam_5985	RNA-dependent DNA polymerase
vFam_69	DNA polymerase
vFam_37	Reverse transcriptase
vFam_387	Capsid protein
vFam_3987	Reverse transcriptase
vFam_3011	Reverse transcriptase
vFam_561	Nucleoprotein N
vFam_1529	Nucleoprotein N
vFam_5143	DNA-directed RNA polymerase subunit alpha
vFam_3906	DNA-directed RNA polymerase subunit alpha
vFam_571	RNA-dependent RNA polymerase
vFam_1961	RNA-dependent RNA polymerase
vFam_1272	RNA-dependent RNA polymerase
vFam_87	Capsid protein
vFam_1118	Capsid protein
vFam_254	DNA polymerase

*`vFAM` database of profile HMMs (http://derisilab.ucsf.edu/software/vFam/).

Table S6. Functional annotation of the contigs obtained by progressive assembly using `vFam` models as seeds.

vFam seed	Number of assembled contigs	Function annotation of the coded protein
vFam_2927	4	Contig 1 – RNA polymerase 2
vFam_2543	1	Contig 1 – DNA-dependent RNA polymerase
vFam_5985	71	Contig 1 – Reverse transcriptase
		Contig 3 – RNA-dependent DNA polymerase
vFam_69	8	Contig 1 – DNA polymerase subunit epsilon
		Contig 2 – DNA polymerase delta
vFam_37	66	Contig 1 – Reverse ribonuclease integrase
vFam_387	18	Contig 1 – Reverse transcriptase
		Contig 2 – DNA helicase
vFam_3987	34	Contig 1 – Reverse ribonuclease integrase
vFam_3011	40	Contig 1 – Reverse ribonuclease integrase
vFam_5143	7	Contig 1 – DNA-dependent RNA polymerase
vFam_3906	5	Contig 1 – DNA-dependent RNA polymerase
vFam_571	1	Contig 1 – RNA-dependent RNA polymerase
vFam_1961	3	Contig 1 – RNA-dependent RNA polymerase
vFam_1272	3	Contig 1 – RNA-dependent RNA polymerase
vFam_87	2	Contig 1 – Capsid protein
vFam_1118	2	Contig 1 – Capsid protein
vFam_254	5	Contig 1 – DNA polymerase
vFam_561	12	Contig 1 – Nucleoprotein N
vFam_1529	6	Contig 1 – Nucleoprotein N

Table S7. Use of UGA and UGG codons in coding sequences of some viruses and their putative hosts.

Accession number	Organism/source	# of UGA / UGG	% of UGA / UGG	AT content (%)
	Leviviridae			
NC_001426	*Escherichia virus BZ13*	***1/26	3.7/96.3	52.14
NC_001417	*Escherichia virus MS2*	0/23	0.8/100	47.88
NC_000913	*Escherichia coli* str. K-12 substr. MG1655	***167/20886	0/100	50.80
	***Botourmiaviridae*/Ourmia-like viruses**			
MH192988	*Aspergillus fumigatus mitovirus 1*	***1/19	5.0/95.0	45.02
NC_017016	*Aspergillus fumigatus* mitochondrion	68/5	93.2/6.8	74.52
NC_013111	*Cassava virus C*	0/17	0/100	50.07
NC_011065	*Epirus cherry virus*	0/14	0/100	46.42
NC_011068	*Ourmia melon virus*	***1/14	6.7/93.3	48.47
	Narnaviridae			
MK984721	*Aedes japonicus narnavirus 1*	0/11 (RdRp)<br>0/9 (HR)	0/100 (RdRp)<br>0/100 (HR)	40.83
MF194022	*Aedes aegypti* mitochondrion	99/3	97.1/2.9	78.96
MK628543	*Culex narnavirus 1*	0/18 (RdRp)<br>0/7 (HR)	0/100 (RdRp)<br>0/100 (HR)	38.54
GU188856	*Culex quinquefasciatus* mitochondrion	94/5	94.9/5.1	78.14
KF298276	*Ochlerotatus-associated narna-like virus 2*	0/15 (RdRp)<br>0/13 (HR)	0/100 (RdRp)<br>0/100 (HR)	40.24
MT993477	*Ochlerotatus caspius* mitochondrion**	15/0	100/0	70.66
NC_004051	*Saccharomyces 20S RNA narnavirus*	***1/18	5.3/94.7	41.73
NC_004050	*Saccharomyces 23S RNA narnavirus*	***1/20	4.8/95.2	41.02
MK628543	*Saccharomyces cerevisiae* mitochondrion	39/0	100/0	83.87
	Mitoviridae			
NC_004046	*Cryphonectria mitovirus 1*	9/8	52.9/47.1	63.42
AF456838	*Cryphonectria parasitica* mitochondrion**	23/5	82.1/17.9	67.95
NC_026510	*Sclerotinia sclerotiorum mitovirus 1*	10/3	76.9/23.1	60.24
MF444234	*Sclerotinia sclerotiorum mitovirus 1-A2*	8/4	66.7/33.3	63.37
KT283062	*Sclerotinia sclerotiorum* mitochondrion	125/29	81.2/18.8	69.10
NC_004052	*Ophiostoma mitovirus 4*	11/2	84.6/15.4	73.26
KY084297	*Ophiostoma novo-ulmi* mitochondrion**	16/1	94.1/5.9	76.18
BK013136	*Lutzomiya longipalpis mitovirus 1*	11/4	73.3/26.7	69.74
BK013137	*Lutzomiya longipalpis* mitochondrion	99/1	99.0/1.0	78.07
MN539817	*Plasmopara viticola associated mitovirus 56*	11/11	50.0/50.0	57.53
NC_045922	*Plasmopara viticola* mitochondrion	***1/101	1.0/99.0	76.29
KX883554.1	*Shahe narna-like virus*	0/8	0/100	65.34
NC_033064	*Wenling narna-like virus 9*	13/1	92.9/7.1	63.84

*Abbreviations: HR - Hypothetical gene coded in the reverse frame; RdRp – RNA-dependent RNA polymerase.
**Incomplete sequence. ***UGA codon used for signaling translation termination.
