# Supplementary figures and images for: Characterization of a Novel Mitovirus of the Sand Fly Lutzomyia longipalpis Using Genomic and Virus–Host Interaction Signatures
Source: Viruses. 2020 Dec 23;13(1):9. doi: 10.3390/v13010009 (PMC7822452; doi:10.3390/v13010009)

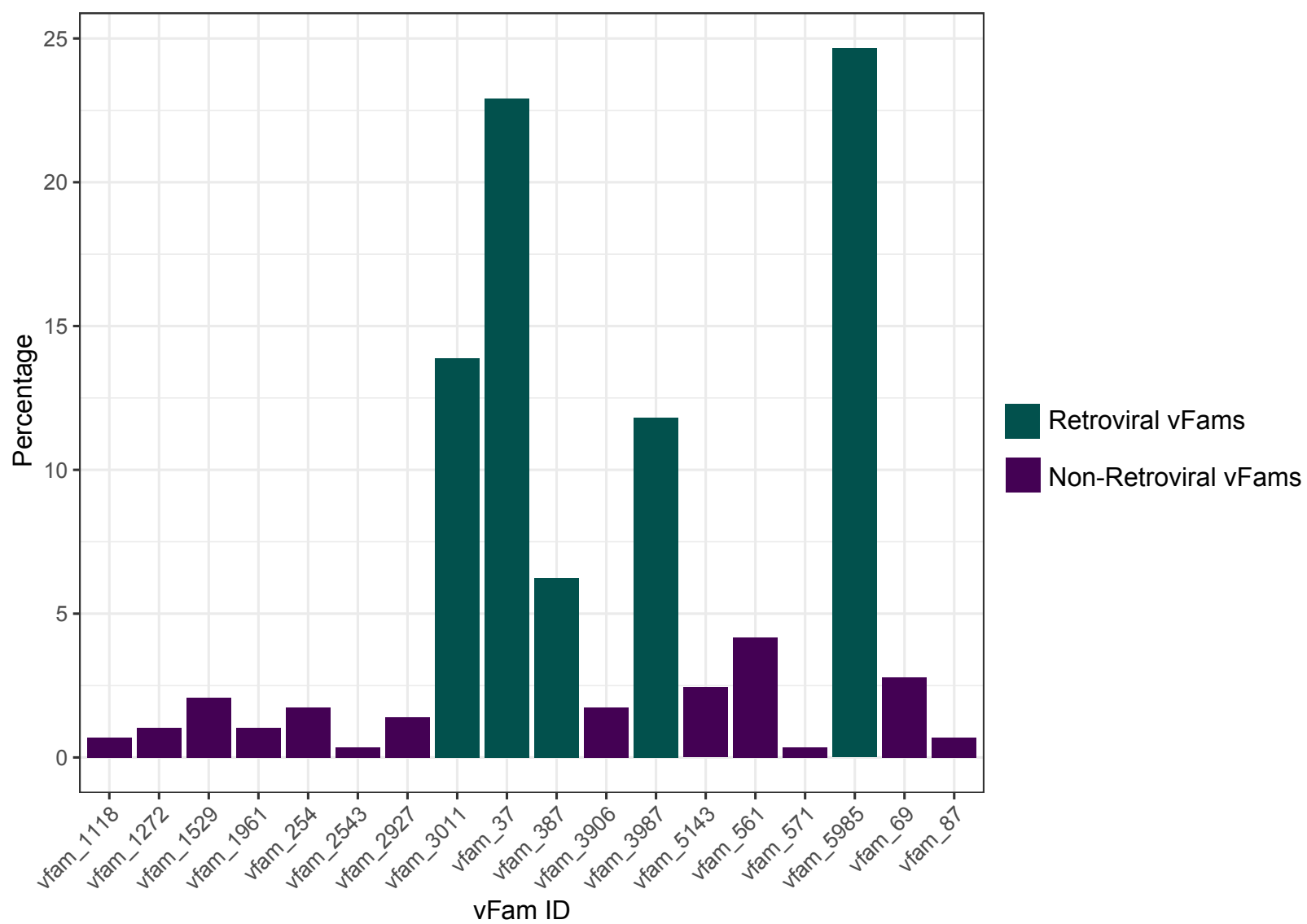

Supplement: Supplementary file 1 [file viruses-13-00009-s001.zip › Figure S1.pdf]

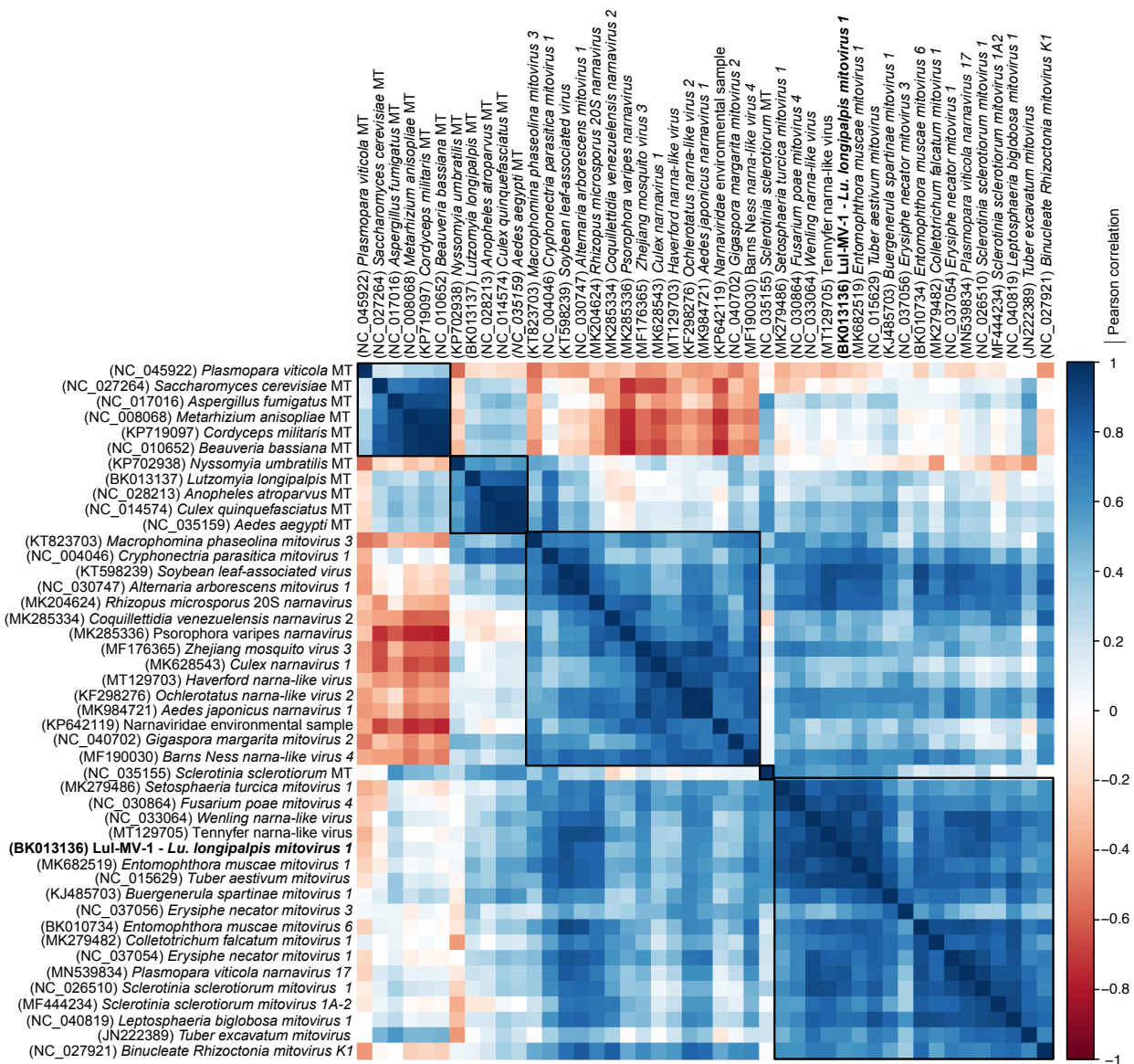

Supplement: Supplementary file 1 [file viruses-13-00009-s001.zip › Figure S2.pdf]

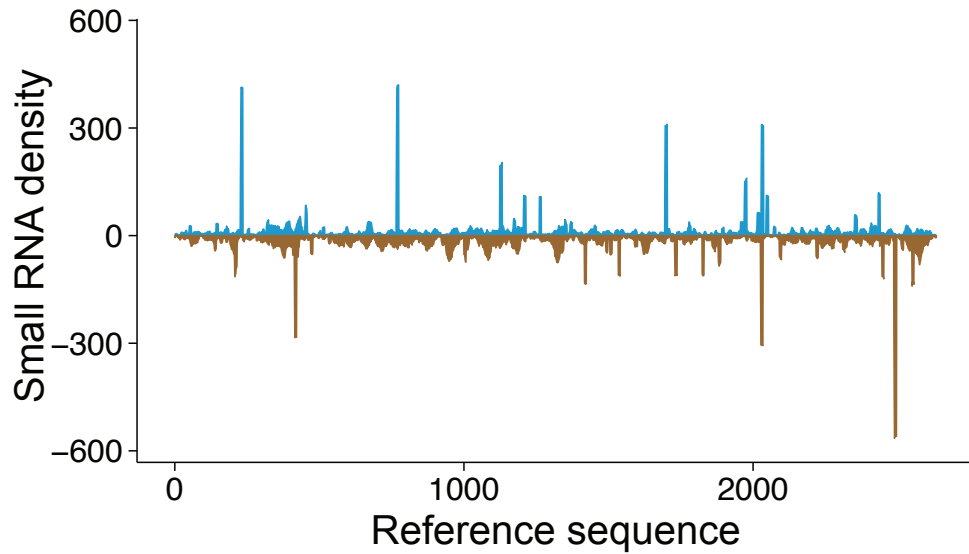

Supplement: Supplementary file 1 [file viruses-13-00009-s001.zip › Figure S3.pdf]
